# The Causes, Counseling, and Prevention Strategies for Maladaptive and Deviant Behaviors in Schools

**DOI:** 10.3390/bs14020118

**Published:** 2024-02-05

**Authors:** Jian-Hong Ye, Mei-Yen Chen, Yu-Feng Wu

**Affiliations:** 1 Faculty of Education, Beijing Normal University, Beijing 100875, China; kimpo30107@hotmail.com; 2 National Institute of Vocational Education, Beijing Normal University, Beijing 100875, China; 3 Graduate Institute of Sport, Leisure and Hospitality Management, National Taiwan Normal University, Taipei 106, Taiwan; meiyen686@gmail.com; 4 Office of Physical Education, Ming Chi University of Technology, New Taipei City 24303, Taiwan

Governments, organizations, and schools around the world are committed to creating a safe and friendly campus environment to ensure students’ high-quality comprehensive development and to cultivate positive mental and physical health states. At the same time, good health and well-being are one of the United Nations sustainable development goals [1]. However, issues such as bullying, truancy, violence, discrimination, and addictive behaviors persist widely in schools around the world. Due to the presence of maladaptive and deviant behaviors among students, educators have been focusing on these issues for the past few decades. However, with the rapid development of internet technology, the impact of school-related incidents has expanded from offline to online and even a mix of online and offline issues. It has become a globally significant public health issue. Maladaptive and deviant behaviors are just factual outcomes; the process of their occurrence and their subsequent effects are also extremely important. Therefore, exploring issues such as the causes of maladaptive and deviant behaviors in the school context, as well as counseling and prevention strategies, is necessary. Moreover, with the global pandemic of COVID-19 during 2020–2023, under the normal pandemic prevention and control, a series of strict student management regulations in colleges and universities could have easily introduced a negative emotional experience [2]. Educational institutions worldwide have undergone changes in their regular routines, affecting both faculty and students. This has resulted in heightened psychological burdens and stress. In addition, psychological depression and anxiety have a negative impact on people’s physical health and daily life [3]. Such circumstances may give rise to latent issues stemming from various direct or indirect factors. As a result, psychological health education, resilience education, and education on overcoming setbacks have received special attention.

In the theme of “The Causes, Counseling, and Prevention Strategies for Maladaptive and Deviant Behaviors in Schools,” there are many research topics worth referencing, including exploration of student engagement, such as the multidimensional model of adolescent school enjoyment; the relationship between student shyness and academic involvement in music; occupational identity and adaptability of engineering students in China; and students’ perceptions of their social participation. Exploration of the impact of stress on academic performance has included a case study on factors influencing secondary school mathematics grades. Exploration of addictive behaviors has included examination of the impact of stress on smartphone addiction) and the effects of short video addiction on students. Exploration of vulnerable student groups has included studies on the impact of parental marital conflict, family socioeconomic status, and depressive symptoms among mobile children) and the perspectives of traumatized children on their life experiences. Additionally, research also includes exploration of adolescent maladaptive behaviors, such as model validation of problem behavior among adolescents, the relationship between harsh parenting and aggressive behavior among male adolescent offenders, and the health-related behaviors and socio-psychological characteristics of smoking adolescents in Korea. Furthermore, some studies have explored issues that also deserve considerable attention, including understanding the role of parent–child relationships in the personality development of Chinese middle school students, the impact of self-flexibility on interpersonal relationship issues among nursing college students, and a literature review of the long-term social withdrawal (hikikomori) phenomenon. These studies have filled the gaps in related research topics, helping people better understand some situations within the teacher and student communities. However, despite numerous studies reviewing or confirming factors or models related to maladaptive and deviant behaviors in schools, cultural differences among countries, regions, and ethnicities, as well as the evolving landscape over time, and technological advancements may lead to variations or the need for updated knowledge regarding many of the established research findings.

Specifically, despite the efforts of educational practitioners to intervene and prevent them, adverse events continue to persist. Moreover, countries and regions worldwide are also committed to relevant research efforts. However, until the issues of maladaptive and deviant behaviors are eradicated, it is necessary to continue in-depth research using various methods such as experimental design, quasi-experimental design, quantitative research, qualitative research, mixed methods, literature reviews, and so on. This will involve continued exploration of the fundamental causes and impacts of maladaptive and deviant behaviors, as well as an examination of the effectiveness of various intervention measures over time. Additionally, the models proposed by theories related to maladaptive and deviant behaviors are not fixed. They can be discussed and enhanced through innovation, construction, development, expansion, and integration of different research models, considering the mechanisms of positive and negative processes in various contexts. At the same time, it is important to explore or develop localized theories, models, variables, measurement tools, etc., according to the culture of the country and the region. Furthermore, teachers and institutional managers also need to adjust theoretical models more effectively and put them into practice based on student characteristics, topics, curriculum content, course context, and class schedules.

Additionally, while media use may be beneficial in certain aspects, there is increasing concern over the potentially negative consequences arising from its excessive use [4]. The reason for this consequence may be due to the influence of societal values or cognitive gaps, as students do not realize that this is an addictive behavior [5]. Therefore, issues related to new media addictions such as video addiction, short video addiction, social media addiction, gaming addiction, and so on continue to be topics of ongoing concern, with relevant research continually evolving. However, there is still an urgent need to continue expanding related research because the issues related to addictive behaviors require more effective intervention methods for prevention and improvement. The negative impacts on adolescents have not been fully explored, and approaching the topic from the perspectives of digital literacy or media literacy could be promising research directions.

Furthermore, leadership has traditionally been considered a key factor in crisis management and the enhancement of well-being. Moreover, different leadership styles are associated with the psychological well-being of followers; however, little is known about the relative strength of the relationship between different leadership styles and the psychological well-being of followers [6]. Therefore, exploring leadership as a means to improve or resolve maladaptive and deviant behaviors will also be an important research direction. Future research could delve into the various leadership styles and capabilities of teachers or students, exploring how they can improve maladaptive and deviant behaviors or enhance psychological health and well-being.

Bullying among adolescents is associated with different mental health issues for the victim and perpetrator [7]. Psychological counseling and guidance for both aggressors and victims among students are important research topics. In addition to traditional psychological research, many experts believe that with the maturity of generative artificial intelligence functions, it is possible for artificial intelligence to accompany humans and achieve conversational functions such as listening, chatting, and counseling. However, research on the relationship between artificial intelligence and maladaptive and deviant behaviors is currently quite limited. Existing articles are often opinion papers, and so research based on data or text is urgently needed. In the context of the digital transformation, there are many emerging technologies that have also been found to be useful in enhancing counseling, as well as mental health and well-being, and all of these practical experiences are worth exploring in a systematic way.

Certainly, mechanisms for prevention and intervention in more severe deviant behaviors such as bullying, truancy, violence, discrimination, crime, underage smoking, and underage sexual behaviors also need to be continuously emphasized and explored. Simultaneously, negative variables such as addiction, aggression, anxiety, avoidance, conflict, cognitive bias, depression, excessive cognitive reflection, evasion, fatigue, fear, hostility, involution (neijuan), laziness, loneliness, rejection, selfishness, silence, stress, withdrawal, and worry should also continue to be discussed. Engagement, gratitude, fairness, flow, hope, kindness, meaning, mindfulness, moral sense, perceived value, positive mindset, relationships, respect, resilience, self-efficacy, self-regulation, sense of happiness (well-being), socio-emotional skills, social support, trust, and other relatively neutral and positive variables can also be explored for their impact mechanisms alongside the negative variables. In summary, ongoing research in the above aspects in the future will contribute to detailed and up-to-date explanations of the measures adopted by governments, organizations, educators, and communities globally to address and prevent maladaptive and deviant behavior in schools. Therefore, we encourage scholars interested in this research topic to explore the causes, consequences, and effective intervention measures for maladaptive and deviant behaviors in schools from different theoretical perspectives within the field of psychology, such as positive psychology, health psychology, educational psychology, counseling psychology, applied psychology, and others.

## References

[1] United Nations Sustainable Development Goals. https://www.un.org/sustainabledevelopment/zh/sustainable-development-goals/.

[2] Chen Y.N. (2023). The Relationship between Personality Traits, Emotional Stability and Mental Health in Art Vocational and Technical College Students during Epidemic Prevention and Control. Psychol. Res. Behav. Manag..

[3] Yu J.E., Eun D., Jee Y.S. (2022). Daily life patterns, psychophysical conditions, and immunity of adolescents in the COVID-19 era: A mixed research with qualitative interviews by a quasi-experimental retrospective study. Healthcare.

[4] Ye J.H., Wu Y.T., Wu Y.F., Chen M.Y., Ye J.N. (2022). Effects of short video addiction on the motivation and well-being of Chinese vocational college students. Front. Public Health.

[5] Lin I.T., Shen Y.M., Shih M.J., Ho C.C. (2023). Short Video Addiction on the Interaction of Creative Self-Efficacy and Career Interest to Innovative Design Profession Students. Healthcare.

[6] Montano D., Schleu J.E., Hüffmeier J. (2023). A meta-analysis of the relative contribution of leadership styles to followers’ mental health. J. Leadersh. Organ. Stud..

[7] Varela J.J., Sánchez P.A., De Tezanos-Pinto P., Chuecas J., Benavente M. (2021). School climate, bullying and mental health among Chilean adolescents. Child Indic. Res..

